# Validating the falls decision rule: optimizing head CT use in older adults with ground-level falls

**DOI:** 10.1007/s43678-025-00937-y

**Published:** 2025-05-13

**Authors:** Emre Kudu, Mustafa Altun, Faruk Danış, Sinan Karacabey, Erkman Sanri, Arzu Denizbasi

**Affiliations:** 1 Department of Emergency Medicine, Marmara University School of Medicine, Fevzi Çakmak Mahallesi, Muhsin Yazıcıoğlu Caddesi, Pendik, Istanbul, Turkey; 2 Department of Emergency Medicine, Bolu Abant İzzet Baysal University Medical School, Bolu, Turkey

**Keywords:** Clinical decision rule, Computed tomography, Falls rule, Frailty, Geriatrics, Intracranial bleeding, Règle de décision clinique, tomographie par ordinateur, Règle des chutes, fragilité, gériatrie, saignement intracrânien

## Abstract

**Objective:**

Falls are a leading cause of traumatic brain injury in older adults, with ground-level falls being the most common mechanism. Despite the increasing use of head computed tomography (CT) in older adults with ground-level falls, there is an ongoing debate regarding the necessity of routine neuroimaging in all cases. The falls decision rule was developed to safely exclude clinically important intracranial bleeding without head CT in older adults. This study aims to validate the falls decision rule externally and assess its accuracy in identifying low-risk patients while reducing unnecessary imaging.

**Methods:**

This prospective cohort study at a Level-1 trauma center enrolled consecutive patients aged ≥ 65 years presenting within 48 h of a ground-level fall. Patient management, including the decision to perform head CT, was determined independently by the treating emergency physician. Patients were followed up for 42 days to identify clinically important intracranial bleeding cases. The rule’s diagnostic performance was evaluated using sensitivity, specificity, and predictive values using 95% confidence intervals (CI).

**Results:**

A total of 800 patients were included, with a median age of 78 years (IQR 72–85), and 59.9% were female. Clinically important intracranial bleeding was identified in 6.1% (*n* = 49) of patients. Head CT was performed in 67.6% of cases, identifying 43 initial hemorrhages, with six additional cases detected during follow-ups. The falls decision rule demonstrated 97.9% sensitivity (95% CI 89.1–99.9), 31.9% specificity (95% CI 28.6–35.4), and 99.5% negative predictive value (95% CI 97.1–99.9), potentially reducing CTs by one-third.

**Conclusion:**

This validation confirms the falls decision rule’s high sensitivity and negative predictive value for identifying low-risk older adults after ground-level falls, potentially reducing unnecessary CT scans by approximately one-third. This approach could alleviate ED overcrowding and resource strain while ensuring diagnostic safety.

**Supplementary Information:**

The online version contains supplementary material available at 10.1007/s43678-025-00937-y.

## Résumé

**Objectifs:**

Les chutes sont la principale cause de traumatismes cérébraux chez les personnes âgées, le mécanisme le plus courant étant les chutes au niveau du sol. Malgré l’utilisation croissante de la tomodensitométrie crânienne (TDM) chez les personnes âgées qui ont des chutes au niveau du sol, il y a un débat en cours sur la nécessité d’une neuroimagerie de routine dans tous les cas. La règle de décision de Falls a été élaborée pour exclure en toute sécurité les saignements intracrâniens cliniquement importants sans TDM de la tête chez les personnes âgées. Cette étude vise à valider la règle de décision des chutes à l’externe et à évaluer sa précision pour identifier les patients à faible risque tout en réduisant l’imagerie inutile.

**Méthodes:**

Cette étude de cohorte prospective dans un centre de traumatologie de niveau 1 a inclus des patients consécutifs âgés de 65 ans présentant une chute au sol dans les 48 heures. La prise en charge du patient, y compris la décision d’effectuer une TDM de la tête, a été déterminée de façon indépendante par le médecin urgentiste traitant. Les patients ont été suivis pendant 42 jours pour identifier des cas de saignements intracrâniens cliniquement importants. La performance diagnostique de la règle a été évaluée à l’aide des valeurs de sensibilité, de spécificité et de prédiction à l’aide d’intervalles de confiance (IC) à 95 %.

**Résultats:**

Un total de 800 patients ont été inclus, dont l’âge médian était de 78 ans (IQR : 72-85), et 59,9 % étaient des femmes. Une hémorragie intracrânienne cliniquement importante a été identifiée chez 6,1 % (n=49) des patients. La TDM de la tête a été effectuée dans 67,6 % des cas, ce qui a permis d’identifier 43 hémorragies initiales et six autres cas ont été détectés au cours du suivi. La règle de décision de Falls a fait preuve d’une sensibilité de 97,9 % (IC à 95 % 89,1-99,9), d’une spécificité de 31,9 % (IC à 95 % 28,6-35,4) et d’une valeur prédictive négative de 99,5 % (IC à 95 % 97,1-99,9), ce qui pourrait réduire les TC d’un tiers.

**Conclusion:**

Cette validation confirme la haute sensibilité et la valeur prédictive négative de la règle de décision des chutes pour identifier les personnes âgées à faible risque après une chute au niveau du sol, ce qui pourrait réduire d’environ un tiers le nombre de tomodensitométries inutiles. Cette approche pourrait atténuer le surpeuplement et la contrainte en matière de ressources des DE tout en assurant la sécurité du diagnostic.

## Clinician’s capsule

**Table:** 

***What is known about the topic?***
Emergency physicians frequently perform head CT scans for older adults with ground-level falls to rule out intracranial bleeding.
***What did this study ask?***
Does the Falls Decision Rule accurately identify low-risk older adults after ground-level falls, reducing unnecessary head CT scans?
***What did this study find?***
The Falls Decision Rule demonstrated 98.0% sensitivity and 99.6% negative predictive value, potentially reducing unnecessary CT scans by one-third, applicable regardless of antithrombotic medication status.
***Why does this study matter to clinicians?***
Implementing the Falls Decision Rule can optimize CT use, alleviate ED overcrowding, and improve resource allocation while maintaining diagnostic safety.

## Introduction

Falls significantly impact older adults, with 28% of those aged ≥ 65 years in the United States experiencing at least one fall annually [1]. Ground-level falls are predominant (69.6%) and constitute a leading cause of traumatic brain injury in this population [2]. Due to high morbidity and mortality in older falls, timely recognition and appropriate management are a critical priority in emergency care. A mainly debated issue in this context is whether all older patients presenting to the ED following falls from ground-level should routinely undergo computed tomography (CT) of the brain [3].

Several validated clinical decision tools, such as the NEXUS II, the New Orleans Criteria, and the Canadian CT Head Rule, have been established to guide imaging decisions in patients with head trauma [4–7]. However, these tools were primarily designed for general head injuries rather than the specific context of ground-level falls in older adults. These rules often recommend routine CT imaging for older patients due to heightened injury risk, an approach reinforced by studies advocating universal scanning in this population [2, 8]. This conservative strategy, while reducing missed diagnoses, increases radiation exposure, costs, and prolonged length of stay, which may lead to ED overcrowding and adverse events in older adults [9–12]. Therefore, algorithms are needed to ensure safe management of the older population.

Recognizing this gap, de Wit et al. developed the falls decision rule for older adults after ground-level falls, incorporating frailty alongside predictors like head injury absence, amnesia, and neurologic abnormalities [13]. In a study of over 4,000 patients, the rule demonstrated high sensitivity (98.6%) in excluding clinically important intracranial bleeding while maintaining a negative predictive value, reducing unnecessary CT scans by 19.9% without compromising safety [10]. However, external validation is needed to confirm its generalizability as emphasized by the authors [10]. To this purpose, our study intends to validate the falls decision rule in an independent cohort and test its applicability and reliability in different healthcare settings.

## Methods

### Study design and settings

This prospective cohort study was conducted at a level-1 trauma center with approximately 200,000 annual ED visits. University Clinical Research Ethics Committee approved the study protocol (protocol number: 09.2024.48, date: 12.01.2024), and the research adhered to the Declaration of Helsinki.

In reporting this study, the STARD guidelines were followed [14], and this study was registered as a clinical trial under the identifier NCT06525727.

### Study population

This study prospectively enrolled all consecutive patients aged ≥ 65 years presenting to the ED within 48 h of a ground-level fall (e.g., from standing, toilet, chair, or bed) between January 20, 2024 and October 1, 2024, regardless of head impact, as in the derivation study. Exclusions included prior study enrollment, discharge against medical advice, incomplete data, transfers from other facilities, or lack of national database registration (precluding follow-up), or refusal to participate in the study.

### Patient assessment and data collection

This observational study was conducted devoid of any external intervention. Each patient included in the investigation was managed and treated by their primary emergency physician, who autonomously determined the necessity for a head CT at the time of presentation.

Detailed patient assessment and data collection are in supplementary material [10, 13, 15, 16].

### Outcome definition and test methods

Since this is an external validation study, the outcome definitions used in the validation study were applied precisely as originally defined. The definition of clinically important intracranial bleeding was defined to include any intracranial bleeding requiring medical or surgical intervention within the 42-day follow-up period or resulting in death. Medical intervention was defined as either temporary or permanent cessation of antiplatelet or anticoagulant therapy, administration of antifibrinolytics, reversal of anticoagulation, hospital admission, or surgical procedures [10]. For brevity and readability, clinically important intracranial bleeding will be referred to as 'intracranial bleeding' within the text.

### Index test (the falls decision rule and the focused falls decision rule)

This study assessed two clinical decision instruments: the falls decision rule and the focused falls decision rule. According to the falls decision rule, a head CT scan is deemed unnecessary if all of the subsequent criteria are satisfied: (1) the patient did not hit their head during the fall as determined through patient history or witness accounts; (2) no novel abnormalities detected on neurological examination; (3) the patient retains memory of the events surrounding the fall; and (4) the clinical frailty scale score is below 5.

The focused falls decision rule, a simplified variant of the original tool, includes only the first two criteria: the absence of head impact during the fall and no new neurological abnormalities upon examination.

### Sample size

The sample size for this diagnostic accuracy study was calculated to validate the falls decision rule. Based on de Wit et al.’s metrics, the minimum required sample size was 663, assuming 80% power and a 0.05 margin of error [10]. To account for potential data loss and measurement errors, the target sample size was increased by 15%, resulting in a minimum of 763 participants [17].

### Statistical analysis

Statistical analyses were conducted using IBM SPSS Statistics for Windows, version 23.0 (IBM Corp., Armonk, NY). Data distribution was evaluated using histograms. Categorical variables were presented as numbers and percentages, while continuous variables were presented as median with interquartile ranges. Chi-squared test was used for categorical variables for comparisons between groups, and Mann––Whitney *U* test was applied for continuous variables. The threshold for statistical significance was established at *p* < 0.05. 

The diagnostic accuracy of the falls decision rule was evaluated by calculating sensitivity, specificity, positive predictive value, negative predictive value, positive likelihood ratio, and negative likelihood ratio, each accompanied by 95% CI.

## Results

**Fig. 1:**
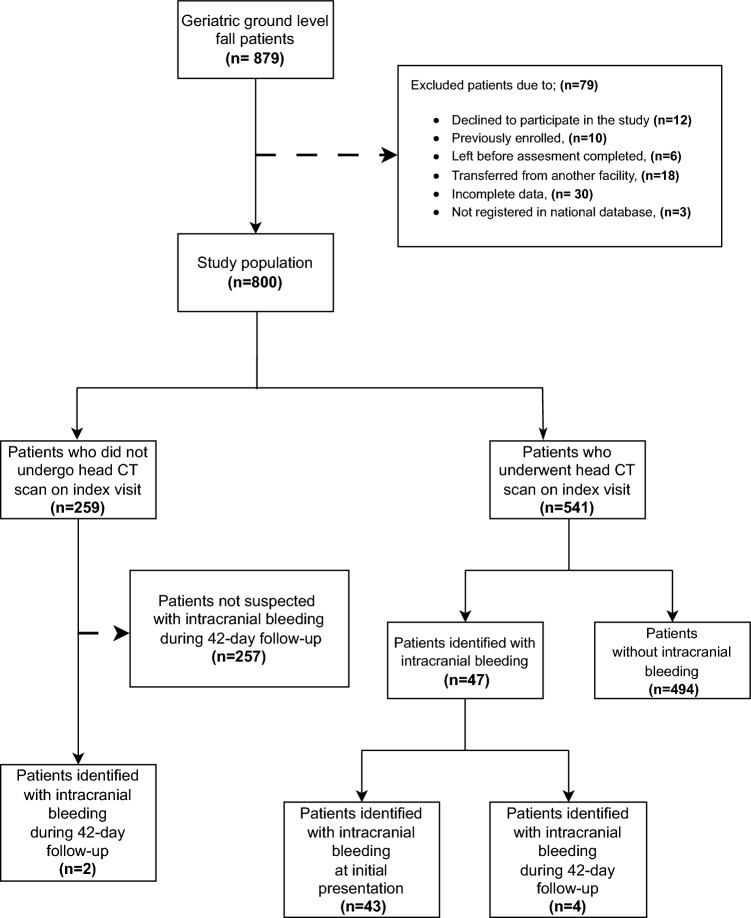
Flow diagram of the study

During the study timeframe, 879 older (≥ 65 years) patients with ground-level falls were assessed, of whom 79 were excluded, resulting in the enrollment of 800 patients (Fig. 1).

The median age of patients in the study cohort was 78 years (IQR 72–85), and 59.9% (*n* = 479) were women. A comparative analysis of baseline characteristics between patients with and without intracranial bleeding is shown in Table 1.

**Table 1:** Baseline demographic and clinical characteristics of patients classified by intracranial bleeding

Variables	All patients (*n* = 800)	Patients without intracranial bleeding (*n* = 751)	Patients with intracranial bleeding (*n* = 49)
Age (year), median (IQR)	78.0 (72.0–85.0)	78.0 (72.0–84.0)	80.0 (71.5–88.0)
Gender, female, %*	59.9	60.9	44.9
Confirmed history of head trauma, %* Unclear history of head trauma, %	56.0 4.9	54.2 4.8	83.7 6.1
Confirmed history of disorientation, %* Unclear history of disorientation, %	6.8 1.1	4.8 0.9	36.7 4.1
Confirmed history of loss of consciousness, %* Unclear history of loss of consciousness, %	5.8 1.4	3.6 1.2	38.8 4.1
Confirmed history of amnesia of events, %* Unclear history of amnesia of events, %	11.1 4.9	9.2 3.6	40.8 24.5
Vomited once, %	11.5	11.3	14.3
Vomited more than once, %	4.0	3.9	6.1
Bruise or laceration on the head, %*	36.4	34.2	69.4
Signs of basal skull fracture, %*	4.5	2.9	28.6
Signs of open or depressed skull fracture, %*	0.9	0.3	10.2
Glasgow Coma Scale, median (IQR)*	15 (15–15)	15 (15–15)	14 (13–15)
Abnormal neurological examination from baseline, %*	4.5	2.3	38.8
Retrograde amnesia for > 30 min, %*	5.5	4.0	28.6
Clinical Frailty Scale score, %* 1 Healthy, active and very fit 2 No disease symptoms, occasional exercise 3 Controlled medical issues, walk only 4 Symptoms limit activities 5 Need help with daily living activities^ 6 Need help with bathing/dressing 7–8 Completely dependent for everything 9 Terminally ill, life expectancy < 6 months	11.0 22.4 27.6 14.8 8.9 7.8 6.6 1.0	11.3 22.6 28.0 15.0 9.2 7.1 5.9 0.9	6.1 18.4 22.4 10.2 4.1 18.4 18.4 2.0
Renal impairment, %	12.9	13.2	8.2
Antiplatelet therapy, %	38.3	37.8	44.9
Anticoagulant therapy, %	12.3	12.0	16.3
Hemoglobin, g/L, median (IQR)	12.0 (10.6–13.3)	12.0 (10.7–13.2)	11.9 (10.4–13.6)
Platelet count × 10^9^/L, median (IQR)	228 (186–271)	230 (186–273)	206 (168–255)
Head CT scan at initial visit, %*	67.6	65.8	95.9
Mortality, %*	5.8	4.5	24.5

^*^ Indicates significant difference between groups, ^ shopping, finances, transportation, heavy housework. *IQR* Interquartile range

Hemoglobin and platelet count data were unavailable for 276 patients

A head CT scan was performed in 67.6% (*n* = 541) of the patients at the initial presentation. Among this group, 43 patients were diagnosed with intracranial bleeding on their initial imaging. Four patients whose initial CT scans showed no abnormalities were later diagnosed with intracranial bleeding during the follow-up period. Of the 259 patients who did not undergo CT imaging at the initial visit, two were identified with intracranial bleeding during follow-up (Fig. 1).

A total of 49 patients had intracranial bleeding, primarily subdural (51.0%), subarachnoid (42.8%), and intraparenchymal (18.3%). Two patients died before head CT could exclude intracranial bleeding, so the possible type of bleeding could not be determined (supplementary material). Neurosurgical intervention was performed in 12.2% (*n* = 6) of the intracranial bleeding cases, and the overall mortality rate of intracranial bleeding cases within the follow-up period was 24.5% (*n* = 12) (Table 2).

**Table 2:** Distribution of intracranial bleeding Sites, neurosurgical Interventions, and mortality in patients with intracranial bleeding

Variable*	n (%)
Bleed site Subdural bleed Subarachnoid bleed Intraparenchymal bleed Intraventricular bleed Epidural bleed Undetermined^#^	25 (51.0) 21 (42.8) 9 (18.3) 3 (6.1) 3 (6.1) 2 (4.0)
Neurosurgical intervention	6 (12.2)
Mortality	12 (24.5)

^*^Patients with multiple injuries were recorded distinctly for each injury sustained and may be represented multiple times within the table. ^#^ Detailed information about these two patients is presented in the supplementary material

A detailed comparison of whether head CT imaging was recommended based on the falls decision rule and focused falls decision rule, alongside the presence of intracranial bleeding, is presented in Table 3. The falls decision rule misclassified one case of intracranial bleeding as not requiring head CT while achieving a sensitivity of 98.0% (95% CI 89.2–100.0) and a negative predictive value of 99.6% (95% CI 97.2–99.9) with a specificity of 32.0% (95% CI 28.6–35.4). This specificity indicates that the rule correctly identified approximately one-third of patients (32.0%, or 240 of 751) as low-risk, potentially avoiding unnecessary CT scans in this group. Although the focused falls decision rule exhibited a higher specificity of 40.9% (95% CI: 37.3–44.5), it misclassified three intracranial bleeding cases and showed a lower sensitivity of 93.9% (95% CI 83.1–98.7) and a negative predictive value of 99.0% (95% CI 97.1–99.7) (Table 3). Details of patients misclassified by the falls decision rule and the focused falls decision rule are presented in supplementary material.

**Table 3:** Diagnostic performance of the falls decision rule and focused falls decision rule

	Patients with intracranial bleeding	Patients without intracranial bleeding	Sensitivity (95% CI)	Specificity (95% CI)	NPV (95% CI)	PPV (95% CI)	NLR (95% CI)	PLR (95% CI)
Falls decision rule			98.0 (89.2–100.0)	32.0 (28.6–35.4)	99.6 (97.2–99.9)	8.6 (8.1–9.1)	0.1 (0.0–0.4)	1.4 (1.4–1.5)
CT indicated	48	511						
CT not indicated	1	240						
Focused falls decision rule			93.9 (83.1–98.7)	40.9 (37.3–44.5)	99.0 (97.1–99.7)	9.4 (8.6–10.2)	0.2 (0.1–0.5)	1.6 (1.5–1.7)
CT indicated	46	444						
CT not indicated	3	307						

*CI* Confidence interval, *CT* Computed tomography, *NLR* Negative likelihood ratio, *NPV* Negative predictive valıe, *PLR* Positive likelihood ratio, *PPV* Positive predictive value

## Discussion

### Interpretation of Findings

This study provides an external validation of the falls decision rule, which was developed to safely exclude intracranial bleeding in older patients who experience ground-level falls without requiring head CT imaging. Our findings confirm that the falls decision rule maintains a high sensitivity and negative predictive value, effectively identifying patients at low risk for intracranial bleeding. Applying this rule could also reduce unnecessary head CT scans in nearly one-third of cases without compromising patient safety. Although the focused falls decision rule has a higher specificity, it is more likely to miss patients due to its lower sensitivity. Therefore, we recommend the falls decision rule as a safer and more reliable option for widespread clinical use. The focused falls decision rule may be considered in resource-limited settings where reducing imaging volume is a priority, but its use should be approached with caution given the potential for missed diagnoses.

### Comparison to previous studies

In our study, while female patients constituted most of those presenting with falls, intracranial bleeding was more frequently observed in male patients. This finding has been explored in multiple studies and has been attributed to various multifactorial causes [18]. De Wit et al. deliberately chose not to include sex as a criterion in their decision tool, reasoning that a head CT assessment algorithm should apply to both genders without introducing potential bias [10]. However, when evaluating ground-level fall patients, it is important to recognize that male patients may be more vulnerable to severe injuries.

In a study by Sartin et al., 437 patients with ground-level falls who underwent head CT were analyzed, revealing that CT findings altered patient management in 21.7% of cases. Based on these results, the authors concluded that no single predictive factor was sufficient to exclude intracranial injury reliably and thus recommended performing CT scans for all patients with ground-level falls [8]. When evaluating older adults, determining the presence of head trauma, new neurological deficits, or amnesia is often challenging. Cognitive impairment, communication barriers, and baseline functional limitations can complicate clinical assessment. While Sartin et al. aimed to identify a single predictor but could not find one de Wit et al. developed a decision rule that integrates a combination of multiple parameters, notably emphasizing frailty as a key component [10]. This addition is particularly valuable in older adults with limited ability to express their symptoms and dependent on others for care. Including frailty as a predictive factor contributed to rule achieving high sensitivity and a substantial negative predictive value, ensuring safe risk stratification even in more vulnerable older populations [19]. Our findings reinforce the relevance of frailty assessment and validate its role in risk stratification.

### Strengths and limitations

The major strength of our study is its prospective cohort design, which ensured systematic data collection from consecutive older adults presenting with ground-level falls, minimizing recall bias. A complete 42-day follow-up was achieved for all patients using Türkiye’s comprehensive healthcare system (e-pulse), enabling the accurate ascertainment of outcomes. Additionally, by externally validating the falls decision rule and focused falls decision rule in a Turkish population, distinct from the original Canadian cohort, we enhance the generalizability and applicability of these tools across diverse settings.

This research encompasses various limitations. First, the single-center study design at a level-1 trauma center may limit the generalizability of our findings to broader healthcare settings. Another limitation is that physician decision-making regarding CT use may vary as previous studies have shown inconsistencies in imaging decisions among emergency physicians [20]. Moreover, it is noteworthy that not every patient received CT imaging upon presentation, suggesting that certain instances of intracranial bleeding might have been missed. However, all patients were followed for 42 days to mitigate these limitations. Similarly, during follow-ups, two patients died, and despite interviews with family members, the exact cause of death could not be definitively determined. These cases were conservatively classified as intracranial bleeding due to their high-risk profile as they would have required CT imaging to rule out intracranial bleeding had they reached the hospital. While this assumption may overestimate intracranial bleeding incidence, it aligns with the study’s safety-focused approach and is unlikely to represent a significant misclassification.

### Clinical implications

The increasing reliance on CT imaging in older fall patients has been well-documented. Brinjikji et al. demonstrated a sharp rise (60%) in head CT utilization over the past two decades despite a relatively low incidence of clinically significant findings [21]. This trend reflects a growing concern over missed diagnoses and medicolegal consequences, leading to unnecessary imaging [21, 22]. However, this practice brings many problems, such as overcrowding of the ED, increased healthcare costs, and potential risks related to radiation [10, 23]. Although radiation-induced harm is less of a concern in older adults due to their shorter life span, prolonged ED stays, resource overutilization, and the risk of delirium remain critical challenges in older patients care. These factors underscore the need for high-sensitivity clinical decision tools to guide physicians in making safer and more efficient imaging decisions while reducing defensive medicine practices. The falls decision rule provides a potential solution by allowing clinicians to more accurately target CT use to high-risk patients while reducing unnecessary imaging. Consistent with de Wit et al.'s findings, we observed that the rule could reduce unnecessary CT scans by approximately 31.9%, demonstrating its potential to optimize resource utilization without compromising patient safety. Another important clinical consideration is that intracranial bleeding can be missed on initial assessment even when CT is performed. In our study, four patients who initially had negative CT scans were later diagnosed with intracranial bleeding during follow-ups, reinforcing the importance of clear discharge instructions. Patients or relatives should be educated about the risk of delayed hemorrhage and advised to return if they experience new neurological symptoms.

### Research implications

The sole patient misclassified by the falls decision rule in our study had a medical history notable for three key factors: antiplatelet use, anticoagulant use, and renal impairment (supplementary material). Although our data did not demonstrate a statistically significant link between antithrombotic therapy alone and intracranial bleeding risk, the coexistence of specific clinical factors—particularly potential drug interactions and renal dysfunction—may point to an underappreciated high-risk profile in this population. Future studies should therefore examine the intricate relationships between antithrombotic treatments and comorbidities such as renal impairment to refine risk stratification and bolster clinical decision-making for older adults who experience falls. Moreover, cost-effectiveness analyses could shed light on the economic advantages of minimizing unnecessary imaging while weighing these benefits against the potential consequences of undetected intracranial injuries.

## Conclusion

In this external validation study, the falls decision rule demonstrated high sensitivity and negative predictive value in identifying older adults at low risk for intracranial bleeding following ground-level falls. Importantly, classifying 32.0% of patients as low-risk could reduce unnecessary CT imaging by approximately one-third, thereby minimizing ED overcrowding, resource overuse, and prolonged ED stays while maintaining diagnostic safety. Considering the growing focus on evidence-informed imaging protocols within trauma management in older patients, we believe this decision-making guideline can enhance clinical judgment and elevate the care of older individuals. 

## Supplementary Information

Below is the link to the electronic supplementary material.

Supplementary file1 (DOCX 20 KB)

## Data Availability

The datasets generated during and/or analyzed during the current study are not publicly available but are available from the corresponding author at reasonable request.
