# Supplementary material for: Validating the falls decision rule: optimizing head CT use in older adults with ground-level falls
Source: CJEM. 2025 May 13;27(8):629–37. doi: 10.1007/s43678-025-00937-y (PMC12380872; doi:10.1007/s43678-025-00937-y)
Supplement: Supplementary file 1 — Supplementary file1 (DOCX 20 KB) [file 43678_2025_937_MOESM1_ESM.docx]

**Methods**

**Patient assessment and data collection**

Demographic information (e.g., age, sex), history of antiplatelet or anticoagulant utilization, particulars of the fall, and clinical frailty scores were gathered by the trained research assistants through interviews with patients, witnesses, or caregivers, supplemented by ED physician notes and medical records, following consent where applicable [10,13]. Specifically, data on disorientation, amnesia, and loss of consciousness were gathered using these methods. Disorientation was defined as confusion about location, time, or identity, often reflecting disrupted self-awareness or self-location. Amnesia was characterized by partial or complete loss of memory following a fall event, while loss of consciousness was defined as a temporary or prolonged state of unawareness of oneself and one's surroundings [15,16]. When these variables were unclear, due to inability to assess the patient, lack of witnesses, they were marked as "unclear" on the standardized patient form. When the treating physician mandated laboratory testing, results such as hemoglobin and platelet levels were documented. The determination to perform a head CT and the subsequent results were also recorded. Outcomes, including the requirement for surgical intervention and mortality, were tracked.

An ED specialist and a radiologist independently evaluated all head CT scans, which were blinded to the patients' demographic and clinical particulars. In cases of disagreement, a third reviewer (radiologist) assessed the images to achieve consensus.

Patients were observed for delayed intracranial bleeding over 42 days, adhering to the protocol established by the derivation study [10]. During this follow-up interval, any subsequent hospital visits were identified via the national healthcare system (e-pulse), and those who presented to the hospital were assessed for evidence of intracranial bleeding. E-pulse is Türkiye’s national comprehensive electronic health database, centralizing detailed medical records—including medical histories, physical examination findings, laboratory results, diagnoses, imaging studies, and vital status—from both public and private hospitals nationwide. This system provided access to patient records from other hospitals, enabling thorough tracking of ED visits, subsequent care, and outcomes across Türkiye for the 42-day follow-up period. In alignment with the derivation study, patients who did not develop symptoms warranting a hospital visit and did not die during the follow-up period were considered negative for intracranial bleeding. Given that previous studies have demonstrated the poor sensitivity (37%, 95% confidence interval [CI] 21-56%) of patient-reported intracranial bleeding, patients who neither revisited the hospital nor died were not actively contacted, adhering to the original derivation protocol [10]. In the event of patient death, the cause of death was determined through follow-up with family members via telephone and a review of medical records. Death reports and related documentation were also examined. The research team collectively reviewed all available data, and the final cause of death was determined by consensus.

**Statistical methods**

**Data management and missing data**

During statistical analysis, clinical variables recorded as “unclear” (head trauma, loss of consciousness, amnesia, or disorientation) on the data collection form were recategorized as positive to ensure a conservative and comprehensive assessment, consistent with the approach used in the derivation study.

All patients were successfully followed for 42 days, with no patients lost throughout the follow-up interval. Patients who died during follow-up and whose cause of death could not be definitively determined were reviewed collectively by the research team. After careful evaluation of all available clinical data, the research team reached a consensus decision to classify two patients (patients #73 and #149) as having experienced intracranial bleeding, since bleeding could not be definitively excluded; therefore, they were considered to require a head CT.

**Detailed patient information**

**Patient #73**

An 87-year-old male patient presented to the emergency department with a complaint of falling from bed. He reported hitting his head during the fall. On examination, his Glasgow Coma Scale (GCS) was 15, and no newly developed neurological deficits, disorientation, altered consciousness, or amnesia were detected compared to his baseline status. He did not vomit at all. He had no history of renal failure. Physical examination revealed only a laceration at the site of head trauma, with no evidence of scalp hematoma or skull fracture. The patient had no history of antiplatelet or anticoagulant use, and his frailty score was assessed as 6. A brain CT performed in the emergency department showed no abnormalities, and the patient was discharged with appropriate recommendations. However, the following day, his relatives were unable to wake him, prompting emergency medical services to be called. During transport, he developed seizures followed by respiratory and then cardiac arrest. Despite all resuscitative efforts, return of spontaneous circulation was not achieved, and the patient was pronounced dead. Although follow-up imaging was not performed, the clinical course strongly suggests that the patient succumbed to an intracranial hemorrhage.

According to the Falls Decision Rule and Falls-Focused Decision Rule, this patient met the criteria for a CT scan.

**Patient #90**

An 80-year-old female patient presented to the emergency department following a fall that occurred while attempting to sit on a chair. She denied any head trauma associated with the fall. On evaluation, her Glasgow Coma Scale (GCS) score was 15, with no new neurological deficits, disorientation, altered consciousness, or amnesia detected compared to her baseline status. She had no history of vomiting. Her medical history included deep venous thrombosis approximately eight months prior, for which she was prescribed rivaroxaban (15 mg daily). Her clinical frailty score was assessed as 5. Physical examination revealed no external signs of head trauma or other abnormal findings. Nevertheless, the primary physician opted to perform a head CT scan due to the patient’s anticoagulant use, which revealed a subdural hematoma. Consequently, her anticoagulant medication was discontinued, and she was closely monitored. On the fifth day of hospitalization, the patient was discharged without complications. At follow-up, it was noted that anticoagulant therapy was not reinitiated.

The Focused Falls Decision Rule classified her as not requiring a head CT due to no head impact or new neurologic abnormalities in this patient. In contrast, the Falls Decision Rule classified her as requiring a CT based on her clinical frailty scale of 5.

**Patient #149**

A 66-year-old female patient was presented to the emergency department after a fall from her own height while utilizing a walker. She did not hit her head during the fall, and her GCS was 15. No newly developed neurological deficits, disorientation, altered consciousness, or amnesia were detected compared to her baseline status. She had no history of vomiting or renal failure. The patient was not using antiplatelet or anticoagulant medications, and her frailty score was assessed as 6. On physical examination, there were no findings suggestive of intracranial injury. However, she had tenderness over her right forearm, which she had struck during the fall, though her joint movements were regular. A forearm X-ray showed no pathology, and she was discharged with appropriate recommendations. On the seventh day post-discharge, the patient was discovered deceased at her residence by relatives. During this period, no symptoms or history suggestive of a cause of death, such as altered consciousness, an infectious process, chest pain, or shortness of breath, were reported. Although a brain CT scan could not be performed, the possibility of intracranial hemorrhage could not be wholly excluded in this patient; consequently, the research team deemed brain CT indicated due to high suspicion of intracranial bleeding.

The Focused Falls Decision Rule classified her as not requiring a head CT due to no head impact or new neurologic abnormalities in this patient, whereas the Falls Decision Rule classified her as requiring a CT based on her clinical frailty scale of 6.

**Patient #370**

A 73-year-old female patient presented to the emergency department due to a fall from her own height. She did not hit her head during the fall, and her GCS was 15. No newly developed neurological deficits, disorientation, altered consciousness, or amnesia were detected compared to her baseline status. She had no history of vomiting. In her medical history, she had renal impairment, coronary artery disease, and atrial fibrillation. She was regularly taking clopidogrel 75 mg daily and enoxaparin sodium 4000 units twice daily. Her frailty score was assessed as 3. Physical examination revealed no pathological findings suggestive of trauma. However, his primary physician decided to perform a head CT scan, and intraparenchymal hemorrhage was detected. The patient was hospitalized, and her antithrombotic medications were discontinued. On the third day of hospitalization, with no evidence of hemorrhage progression, she was discharged with continued enoxaparin sodium therapy.

Despite the presence of intracranial hemorrhage, the patient was classified by the Falls Decision Rule and Falls-Focused Decision Rule as **not** requiring a CT scan.
